# Stratifying risk of acute kidney injury in pre and post cardiac surgery patients using a novel biomarker-based algorithm and clinical risk score

**DOI:** 10.1038/s41598-019-53349-1

**Published:** 2019-11-18

**Authors:** William T. McBride, Mary Jo Kurth, Gavin McLean, Anna Domanska, John V. Lamont, Daniel Maguire, Joanne Watt, Peter Fitzgerald, Ian Young, Jijin Joseph, Mark W. Ruddock

**Affiliations:** 1 Department of Cardiac Anaesthesia, Belfast Health & Social Care Trust, 274 Grosvenor Road, Belfast, BT12 6BA Northern Ireland UK; 2 Randox Laboratories Ltd, Clinical Studies Group, 55 Diamond Road, Crumlin, County Antrim, BT29 4QY Northern Ireland, UK; 3 School of Medicine, Dentistry and Biomedical Sciences, Queen’s University of Belfast, 97 Lisburn Road, Health Sciences Building, Belfast, BT9 7BL Northern Ireland UK

**Keywords:** Cardiology, Predictive markers

## Abstract

Acute kidney injury (AKI) following cardiac surgery significantly increases morbidity and mortality risks. Improving existing clinical methods of identifying patients at risk of perioperative AKI may advance management and treatment options. This study investigated whether a combination of biomarkers and clinical factors pre and post cardiac surgery could stratify patients at risk of developing AKI. Patients (n = 401) consecutively scheduled for elective cardiac surgery were prospectively studied. Clinical data was recorded and blood samples were tested for 31 biomarkers. Areas under receiver operating characteristic (AUROCs) were generated for biomarkers pre and postoperatively to stratify patients at risk of AKI. Preoperatively sTNFR1 had the highest predictive ability to identify risk of developing AKI postoperatively (AUROC 0.748). Postoperatively a combination of H-FABP, midkine and sTNFR2 had the highest predictive ability to identify AKI risk (AUROC 0.836). Preoperative clinical risk factors included patient age, body mass index and diabetes. Perioperative factors included cardio pulmonary bypass, cross-clamp and operation times, intra-aortic balloon pump, blood products and resternotomy. Combining biomarker risk score (BRS) with clinical risk score (CRS) enabled pre and postoperative assignment of patients to AKI risk categories. Combining BRS with CRS will allow better management of cardiac patients at risk of developing AKI.

## Introduction

Acute kidney injury (AKI) is a major complication following cardiac surgery that can affect the function of multiple organs including the brain, lungs and gut. Acute kidney injury increases the risk of death resulting in major use of hospital resources and elevated costs^1^. Acute kidney injury occurs in almost 30% of patients following cardiac surgery^2^ and 50% in high risk patients i.e. diabetics^3,4^. In the UK, approximately 100,000 deaths per year are linked to AKI. Acute kidney injury costs the NHS between £434 and £620 million per year^5^.

Several diagnostic criteria have been used for diagnosis of AKI including the risk, injury, failure, loss, end-stage renal disease (RIFLE) criteria in 2004^6^; AKI Network (AKIN) modified RIFLE criteria in 2007^7^; 2012 Kidney Disease: Improving Global Outcomes (KDIGO) criteria^8^, which combines RIFLE and AKIN criteria. The KIDGO criteria rely on changes in serum creatinine (SCr) levels and urine output, which is not ideal. Oliguria frequently occurs following cardiac surgery. Sometimes it precedes increased SCr due to renal injury, but often is a physiological response to hypovolemia or hypotension. Thus, the specificity of urine output as a criterion for AKI is low, and if used alone could misclassify non AKI as AKI patients^9^. Moreover, at cardiac surgery, increases in SCr above preoperative baseline due to AKI may take several hours to develop due to the inevitable haemodilution effect of cardiopulmonary bypass. Accordingly, increases in SCr if used as a sole criterion for AKI could delay the time to AKI diagnosis and its potential management.

Existing strategies to prevent or reduce risk of AKI at cardiac surgery include (among others) maintaining a higher haematocrit perioperatively^10^ and supra normal blood pressure throughout the operation using vasopressors. However, the use of blood transfusion is costly and not risk free. Furthermore, maintaining a supra normal blood pressure perioperatively during cardiac surgery may heighten the risk of postoperative bleeding at the operative site with its attending complications. Accordingly, the anaesthesiologist must evaluate the risk/benefit balance of such reno-protective interventions for each patient. An ability to accurately stratify patients into AKI risk categories perioperatively would greatly assist in this decision and allow consideration of preventative strategies. However, in addition to predicting risk of AKI, earlier diagnosis might allow interventions such as earlier renal replacement therapy. In this context, some biomarkers have been evaluated to consider their utility in allowing preoperative prediction or perioperative diagnosis of AKI at cardiac surgery. Some of these studies were limited by small sample numbers and/or reduced areas under receiver operating characteristic (AUROC)^11–14^. McBride *et al*.^15^ have shown in elective cardiac surgery patients that IL-10 in postoperative plasma collected 2 hours after surgery and urinary transforming growth factor beta (TGFβ-1), collected 24 hours after surgery, were significantly higher in patients who developed early renal dysfunction. Furthermore, urinary IL-1Ra and sTNFR2 were significantly lower 24 hours postoperatively in late renal dysfunction patients.

Due to the different processes involved and the dynamic nature of AKI, it is unlikely that one biomarker will predict or diagnose AKI across a wide range of clinical conditions and quantify its severity. Therefore, the aim of this study was to investigate whether a combination of biomarkers and clinical factors could be used to stratify the risk of a patient developing AKI pre and post cardiac surgery earlier than routinely used clinical methods. The biomarkers selected for this study were likely to represent key pathways in AKI pathogenesis namely, inflammation, hypoperfusion and reperfusion (see Table 1).

**Table 1:** Biomarkers and their functional status and pathophysiology

Marker	Functional Status	Pathophysiology
IL-1α	Inflammation	Pro inflammatory cytokine involved in the malfunction, injury, and local inflammation of renal cells^41,42^
IL-1β	Inflammation/Ischemia	Pro inflammatory cytokine involved in the malfunction, injury, and local inflammation of renal cells^25,41^ IL-1β is generated by the injured epithelial proximal tubular cell and is an important mediator of endothelial ischemic injury^43^
IL-2	Inflammation	Pro inflammatory cytokine IL-2 is elevated in haemodialysis patients with uremic pruritus^44^
IL-4	Inflammation	Anti-inflammatory cytokine elevated in end stage renal disease^45^
IL-6	Inflammation/Ischemia	Pro inflammatory cytokine involved in orchestration of the inflammatory response following acute renal insult^46^. Renal IL-6 expression in renal tubular epithelial cells is significantly increased in the pathogenesis of AKI^47^ IL-6 is generated by injured epithelial proximal tubular cells and is an important mediator of endothelial ischemic injury^43^
IL-8	Inflammation	IL-8 is generated by injured epithelial proximal tubular cells and is an important mediator of endothelial ischemic injury^43^
IL-10	Inflammation	IL-10 is an anti-inflammatory cytokine involved in the regulation and maintenance of normal renal function^48^
VEGF	Inflammation	Pro inflammatory growth factor involved in angiogenesis^42^
EGF	Mitogen	Intrarenal EGF expression is decreased in tubular injury; decreased urine EGF excretion is a marker for CKD progression^49^
TNFα	Inflammation	Pro inflammatory cytokine associated with renal disease^42^
IFNγ	Activator of macrophages	Cytokine involved in the pathophysiology of CKD^50^
MCP-1	Inflammation	Pro inflammatory cytokine involved in the pathogenesis of CKD^42^
IGF-1	Growth factor	Serum IGF-1 levels are positively associated with CKD^51^
Eotaxin	Inflammation	Inflammatory marker, the chemokine eotaxin, is a predictor of the incidence of renal failure^52^.
IL-1Ra	Inflammation/Ischemia	Anti-inflammatory cytokine involved in renal ischemic reperfusion injury^53^
PDGF-BB	Growth factor	Growth factor involved in driving renal fibrosis; independent of underlying kidney disease^54^
IP-10	Chemokine	Serum IP-10 is a marker for underlying renal disease^55^
IL-12p40	Inflammation	IL-12p40 is a key pro inflammatory cytokine involved in crescentic glomerulonephritis^56^
sIL2Ra	Inflammation	Inflammatory modulator involved in the progression of interstitial fibrosis in CKD^57^
sIL6R	Inflammation	Pro inflammatory cytokine which is elevated in patients with CKD^58^
sTNFR1	Inflammation	sTNFR1 is associated with kidney disease progression^59^
sTNFR2	Inflammation	sTNFR2 is a marker for kidney tissue damage^60^
MMP9	Inflammation	MMP9 increases the expression of TGF-β1 and promotes the occurrence of renal interstitial fibrosis^61^
NGAL	Ischemia	NGAL is a non-invasive urinary biomarker for renal ischemia^14^
CRP	Inflammation	Marker of inflammation in AKI^62,63^
D-Dimer	Inflammation	D-Dimer levels are elevated in renal insufficiency^64^.
NSE	Enzyme	NSE is elevated in patients who present with kidney disease^65^
H-FABP	Ischemia	H-FABP is a marker for detection of ischaemic injury^33^
MK	Ischemia	After ischaemic reperfusion, MK is up-regulated in the proximal tubules. The absence of MK protects against renal ischaemic reperfusion injury by reducing the infiltration of leukocytes^38^

IL, interleukin; AKI, acute kidney disease; CKD, chronic kidney disease; VEGF, vascular endothelial growth factor; EGF, epidermal growth factor; TNFα, tumour necrosis factor alpha; IFNγ, interferon gamma; MCP, monocyte chemoattractant protein; IGF, insulin-like growth factor; IL-1Ra, interleukin-1 receptor antagonist; PDGF-BB, platelet-derived growth factor beta homodimer; IP-10, interferon gamma-induced protein 10; IL-12p40, interleukin-12 subunit p40; sIL2Ra, soluble interleukin-2 receptor alpha; sIL6R, soluble interleukin 6 receptor; sTNFR1, soluble tumour necrosis factor receptor-1; sTNFR2, soluble tumour necrosis factor receptor-2; MMP9, matrix metallopeptidase 9; TGFβ1, transforming growth factor beta 1; NGAL, neutrophil gelatinase-associated lipocalin; CRP, C-reactive protein; NSE, neuron-specific enolase; H-FABP, heart-type fatty acid-binding protein; MK, midkine.

## Materials and Methods

### Study population

**Figure 1:**
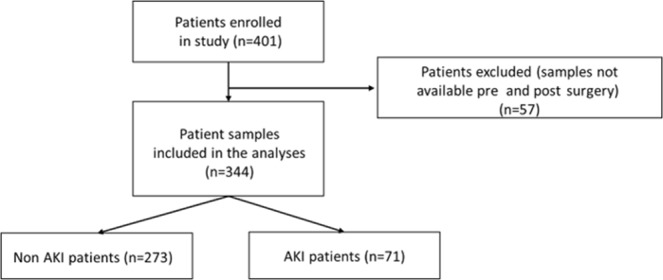
Trial flow diagram.

Cardiac patients who were consecutively scheduled for elective cardiac surgery within the Cardiac Surgical Unit of the Royal Victoria Hospital, Belfast, UK between May 2012 and August 2013, were recruited into the study. Patients were excluded if they were <18 years of age, had preoperative or pretrauma dialysis-dependent renal failure or known significant renal disease. In addition, emergency surgery patients and patients with active malignancy, active endocarditis, sepsis, septic or cardiogenic shock, or had pre-operative haemodynamic instability prior to entrance into the study (known estimated glomerular filtration rate (eGFR < 30)) were excluded. The study complied with the Declaration of Helsinki, was approved by the Office for Research Ethics Committee Northern Ireland, the Royal Victoria Hospital Research Office Research Governance Committee and written informed consent was obtained from all participating patients. The study complied with Standards for Reporting Diagnostic Accuracy (STARD) guidelines^16^. Of the n = 401 patients recruited to the study, pre and postoperative samples were available from 344/401 (85.8%). Patient samples were not available from 57/401 (14.2%) and these patients were excluded from the study (Fig. 1).

### Clinical data collection

Clinical data was recorded for each patient from medical records that included baseline demographic characteristics, comorbidity data and current medications. Creatinine levels in patients pre and post surgery were measured by the hospital laboratory and were used to calculate eGFR through the Modification of Diet in Renal Disease (MDRD) study equation formula^17^. Surgical interventions included: administration of inotropes (e.g. dopamine, adrenaline, milrinone), surgical methods (e.g. valve surgery), cardio pulmonary bypass (CPB) and aortic clamping. Moreover, the length of patient admission (days) in the intensive care unit (ICU) and high dependency unit (HDU), as well as administration of interventions during and postoperatively (e.g. platelets), were also recorded. Clinical and demographic data for each patient were recorded on a case report form and stored on a database.

### Sampling and laboratory methods

Patient blood samples (10 ml) were collected preoperatively and on day 1 postoperatively. The preoperative blood sample was collected following routine arterial line insertion prior to induction of anesthesia. Day 1 (within 24 hours) postoperative blood sample (10 ml) was collected from the same line. Patient blood samples were centrifuged, serum/plasma was aliquoted within 30 minutes of collection and stored at −80 °C.

Patient blood samples were analysed by Randox Clinical Laboratory Services (RCLS) (Antrim, UK) on cytokine arrays (Randox Laboratories Ltd, Crumlin, UK) for the following proteins: Cytokine I Array: Interleukin(IL)-1α, -1β, -2, -4, -6, -8, -10, vascular endothelial growth factor (VEGF), epidermal growth factor (EGF), tumour necrosis factor alpha (TNFα), interferon gamma (IFNγ), and monocyte chemoattractant protein 1 (MCP-1); Cytokine II Array: insulin growth like factor 1 (IGF-1), Eotaxin, interleukin-1 receptor antagonist (IL-1Ra), platelet-derived growth factor beta homodimer (PDGF-BB), interferon gamma induced protein 10 (IP-10), interleukin 12 subunit p40 (IL-12p40); and Cytokine IV Array: soluble interleukin 2 receptor alpha (sIL2Rα), soluble interleukin 6 receptor (sIL6R), soluble tumour necrosis factor receptor 1 (sTNFR1), soluble tumour necrosis factor receptor 2 (sTNFR2) and matrix metallopeptidase 9 (MMP9); using an Evidence Investigator analyser according to manufacturer’s instructions (Randox, Crumlin, UK). Neutrophil gelatinase-associated lipocalin (NGAL), C-reactive protein (CRP), D-Dimer, neuron-specific enolase (NSE), sTNFR1 were measured using Cerebral II Array (Randox, Crumlin, UK). Heart-type fatty-acid binding protein (H-FABP) was measured using an H-FABP IT assay (Randox, Crumlin, UK) on the RX Imola analyser (Randox, Crumlin, UK). Midkine (MK) was measured using a commercial ELISA according to manufacturer’s instructions (CellMid, Sydney, Australia). Serum creatinine was measured in the Kelvin Laboratory (Royal Victoria Hospital, Belfast) on the Cobas 8000c701 (Roche Diagnostics, Basel, Switzerland).

The limits of sensitivity for the biomarkers under investigation were as follows: Cytokine I - IL-2 4.9 pg/ml; IL-4 3.5 pg/ml; IL-6 0.4 pg/ml, IL-8 2.3 pg/ml; VEGF 10.8 pg/ml; IFNγ 2.1 pg/ml; TNFα 3.7 pg/ml; IL1α 0.9 pg/ml; MCP1 25.5 pg/ml; EGF 2.5 pg/ml; IL-10 1.1 pg/ml; IL-1β 1.3 pg/ml; Cytokine II - IL-1Ra 16.83 pg/ml, PDGF-BB 16.16 pg/ml, IP-10 7.81 pg/ml, IL-12p40 7.81 pg/ml; Cytokine IV - sIL2A 0.12 ng/ml; sIL6R 0.62 ng/ml; sTNFR1 0.09 ng/ml; sTNFR2 0.2 ng/ml; MMP9 3.03 ng/ml; CRP 0.67 mg/l; D-Dimer 2.1 ng/ml; NSE 0.26, NGAL 17.8 ng/ml; sTNFR1 0.24 ng/ml; MK 8.0 pg/ml; H-FABP 2.94 ng/ml and SCr 5 μmol/L. Biomarker values below the limit of detection (LOD) were recorded as 90% of LOD.

### Outcome definitions

The development of AKI was defined as an eGFR drop of ≥25% from preoperative baseline on any of the recorded postoperative sampling days (days 1, 2 or 5) or at any time postoperatively.

### Statistical analyses

Statistical analyses were performed using SPSS v25^18^. A Mann-Whitney U Test was used to identify significant biomarkers. Biomarkers with a p < 0.05 were considered significant. The ability of these biomarkers to predict AKI was further investigated using logistic regression (Backward Wald and Forced Entry). Areas Under the Receiver Operator Curve were generated pre and postoperatively for biomarker-based algorithms to provide a measure of how well the biomarker models distinguished between the two diagnostic groups (AKI vs. non AKI). The best combinations of biomarkers (with the greatest AUROC, sensitivity and specificity) were chosen to stratify patients at potential risk of developing AKI.

## Results

A summary of baseline and clinical characteristics of the patients involved in the study are described in Table 2.

**Table 2:** Summary of baseline and clinical characteristics of the study patients.

	non AKI (n = 273)	AKI (n = 71)	p value
**Patient characteristics**			
Age (years)	65.4 ± 11.6	68.6 ± 10.7	0.020
Gender (male)	192/273 (70.3%)	50/71 (70.4%)	0.988
Weight (kg)	80.9 ± 17.5	84.8 ± 16.6	0.061
Height (cm)	167.8 ± 11.4	165.1 ± 14.0	0.082
BMI (kg/m2)	28.9 ± 10.2	31.0 ± 6.0	0.001
**Comorbidities**			
Myocardial Infarction	73/268 (27.2%)	13/68 (19.1%)	0.171
Ischemic Heart Disease	218/268 (81.3%)	65/68 (95.6%)	0.760
Hypertension	35/268 (13.1%)	10/68 (14.7%)	0.722
Diabetes	29/268 (10.8%)	16/68 (23.5%)	0.006
Chronic Obstructive Pulmonary Disease	9/268 (3.4%)	3/68 (4.0 %)	0.676
Diverticulitis	8/268 (3.0%)	3/68 (4.4%)	0.555
Asthma	6/268 (2.2%)	2/68 (2.9%)	0.735
Transient Ischemic Attack	6/268 (2.2%)	1/68 (1.5%)	0.692
Peripheral Vascular Disease	4/268 (1.5%)	2/68 (2.9%)	0.421
Cerebrovascular Accident	4/268 (1.5%)	1/68 (1.5%)	0.989
Endocarditis	1/268 (0.4%)	1/68 (1.5%)	0.294
**Pre surgery medications**			
Beta blockers	186/266 (70.0%)	47/68 (69.1%)	0.897
Calcium antagonists	49/265 (18.5%)	18/68 (26.5%)	0.144
Nitrates	61/265 (23.0%)	13/68 (19.1%)	0.491
Potassium channel blockers	39/265 (14.7%)	8/68 (11.8%)	0.533
ACE inhibitors	131/265 (49.4%)	36/68 (52.9%)	0.606
Angiotensin II blocker	9/265 (3.4%)	2/68 (2.9%)	0.852
**Intraoperative conditions**			
Dopamine	136/267 (50.9%)	40/67 (59.7%)	0.200
Noradrenaline	158/267 (59.2%)	42/67 (62.7%)	0.601
Adrenaline	10/266 (3.8%)	4/67 (6.0 %)	0.421
Milrinone	33/267 (12.4%)	15/67 (22.4%)	0.037
CPB time (min)	132.8 ± 50.7	152.1 ± 61.3	0.018
Cross clamp time (min)	91.7 ± 40.2	105.3 ± 47.6	0.018
Operation time (min)	296.3 ± 125.4	319.7 ± 108.5	0.029
Intra-aortic balloon pump	9/266 (3.4%)	7/67 (10.4%)	0.016
Packed red blood cells	126/266 (47.4%)	41/67 (61.2%)	0.043
Fresh frozen plasma	20/266 (7.5%)	7/67 (10.4%)	0.433
Platelet bags	25/266 (9.4%)	8/67 (11.9%)	0.534
**Operative method**			
Valve Surgery	118/267 (44.2%)	47/68 (69.1%)	<0.001
CABG	178/267 (66.7%)	44/68 (64.7%)	1.000
Valve Surgery + CABG	40/267 (15%)	21/68 (30.9%)	0.002
**Postoperative conditions**			
Dopamine	156/268 (58.2%)	51/67 (76.1%)	0.008
Noradrenaline	163/268 (60.8%)	51/67 (76.1%)	0.023
Adrenaline	12/267 (4.5%)	12/67 (17.9%)	<0.001
Milrinone	39/267 (14.6%)	21/67 (31.3%)	0.001
Packed red blood cells	110/267 (41.2%)	36/67 (53.7%)	0.065
Fresh frozen plasma	39/266 (14.7%)	13/67 (19.4%)	0.340
Platelet bags	39/266 (14.7%)	18/67 (26.9%)	0.018
Resternotomy	11/267 (4.1%)	10/67 (14.9%)	0.001
Readmitted to intensive care	1/267 (0.4%)	0/68 (0.00%)	0.614
Length of admission (days)	11.0 ± 8.0	13.1 ± 7.2	<0.001
Length of ICU admission (days)	2.4 ± 3.0	4.1 ± 3.5	<0.001
Length of stay HDU (days)	1.3 ± 1.0	1.6 ± 1.0	0.001

Data are presented as mean ± standard deviation or number/total (percentages). Note that patients presented with multiple comorbidities. BMI, body mass index; ACE, angiotensin-converting-enzyme; CPB, cardio pulmonary bypass; CABG, coronary artery bypass graft; ICU, intensive care unit; HDU, high dependency unit.

Estimated GFR was recorded on days 1, 2 and 5 following surgery. To increase the number of patients in each cohort, the analyses are based upon an ‘AKI any-day’ definition (i.e. development of AKI on days 1, 2 and 5). Patients were included in this category if their eGFR dropped ≥25% from baseline, following cardiac surgery.

### Preoperative biomarkers

**Figure 2:**
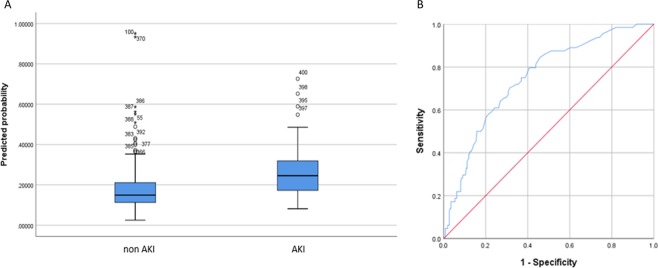
Preoperative biomarker for detecting AKI risk. (**A**) Serum sTNFR1 pre cardiac surgery was significantly higher in patients who developed AKI. (**B**) Soluble TNFR1 had the highest predictive ability to identify patients at risk of developing AKI (AUROC 0.748). AKI, acute kidney injury; sTNFR1, soluble tumour necrosis factor receptor 1; AUROC, area under receiver operating characteristic

Preoperatively sTNFR1 or sTNFR2 had the highest predictive ability to identify patients at risk of developing AKI (Table 3) (sTNFR1 sensitivity 70.3%; specificity 68.5%; AUROC 0.748 (CI 0.684–0.812)) (Fig. 2a,b).

**Table 3:** Serum biomarkers for predicting AKI pre and post cardiac surgery.

	Biomarkers	AUROC	CI	Sensitivity	Specificity
**Anytime**					
Preoperative	sTNFR2	0.713	0.647–0.778	65.6% (42/64)	65.9% (170/258)
	sTNFR1	0.748	0.684–0.812	70.3% (45/64)	68.5% (178/260)
Postoperative	MK	0.704	0.633–0.775	70.7% (41/58)	61.3% (130/212)
	H-FABP	0.729	0.663–0.794	63.1% (41/65)	68.1% (175/257)
	sTNFR2	0.762	0.699–0.825	69.2% (45/65)	69.2% (175/253)
	sTNFR1	0.774	0.708–840.0	72.3% (47/65)	74.0% (188/254)
	H-FABP + MK + sTNFR1	0.817	0.761–0.872	81.0% (47/58)	67.8% (141/208)
	H-FABP + MK + sTNFR2	0.836	0.785–0.888	75.9% (44/58)	69.1% (143/207)

AUROC, Sensitivity and specificity for serum biomarkers for predicting AKI pre and post cardiac surgery.

AKI, acute kidney injury; AUROC, area under receiver operating characteristic; CI, confidence interval; sTNFR2, soluble tumour necrosis factor receptor 2; sTNFR1, soluble tumour necrosis factor receptor 1; MK, midkine; H-FABP, heart-type fatty acid-binding protein.

### Postoperative biomarkers

**Figure 3:**
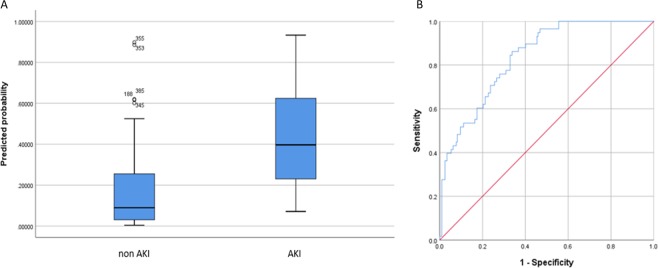
Postoperative biomarker-based algorithm for detecting AKI risk. (**A**) Serum H-FABP, MK and sTNFR2 any time post surgery were significantly higher in patients who developed AKI. (**B**) H-FABP, MK and sTNFR2 had the highest predictive ability to identify patients at risk of developing AKI (AUROC 0.836). AKI, acute kidney injury; H-FABP, heart-type fatty acid-binding protein; MK, midkine; sTNFR2, soluble tumour necrosis factor receptor 2; AUROC, area under receiver operating characteristic

Postoperatively a combination of H-FABP, MK and sTNFR1 or sTNFR2 had the highest predictive ability to identify patients at risk of developing AKI (Table 3) (H-FABP + MK + sTNFR2 sensitivity 75.9%; specificity 69.1%; AUROC 0.836 (CI 0.785–0.888)) (Fig. 3a,b).

### Clinical risk score (CRS)

The main clinical factors identified for patients at potential risk for the development of AKI pre and postoperatively are described in Tables 4 and 5, respectively. Patients who have a cumulative score of 0, no risk. Patients who score >1 e.g. a 65-year-old patient with a BMI of 26 and diabetes would have a cumulative score of 2.5 (highest risk).

**Table 4:** Clinical factors identified for patients at risk of developing AKI pre cardiac surgery and CRS (result).

Clinical Factors	Parameter	Result
Age	<65 ≥65	0 1
BMI	<25 ≥25 <30 ≥30	0 0.5 1
Diabetes	No Yes	0 1

AKI, acute kidney injury; CRS, clinical risk score; BMI, body mass index.

**Table 5:** Clinical factors identified for patients at risk of developing AKI 24 hours post cardiac surgery and CRS (result).

Clinical Factors	Parameter	Result
Age	<65 ≥65	0 1
BMI	<25 ≥25 <30 ≥30	0 0.5 1
Diabetes	No Yes	0 1
CPB time (min)	<130 ≥130	0 1
Cross clamp time (min)	<90 ≥90	0 1
Operation time (min)	<296 ≥296	0 1
Intra-aortic balloon pump	No Yes	0 1
Packed red blood cells	No Yes	0 1
Platelet bags	No Yes	0 1
Resternotomy	No Yes	0 1

AKI, acute kidney injury; CRS, clinical risk score; BMI, body mass index; CPB, cardio pulmonary bypass.

### Biomarker risk score (BRS)

Biomarker combination algorithm(s) can be applied clinically to provide a patient risk score for developing AKI. Patients with a score equal to/or greater than the value of the set point (cut-off) would be categorised positive (AKI); whereas patients below the cut-off would be categorised negative (non AKI) (Table 6).

**Table 6:** Post surgery patient score calculation and BRS.

BRS	Patient score^*^
Negative	<0.200
Positive	≥0.200

*Patient Score = 7.322 + 1.773 *log(H-FABP) + 1.120 *log(MK) + 3.510 *log(sTNFR2).

The patient score equation was derived from logistic regression. The cut-off (0.200) was manually determined to optimise sensitivity while maintaining specificity. If the patient score was < 0.200 then BRS is negative for AKI, if the patient score ≥ 0.200 then BRS is positive for AKI.

BRS, biomarker risk score; H-FABP, heart-type fatty acid-binding protein; MK, midkine; sTNFR2, soluble tumour necrosis factor receptor 2; AKI, acute kidney injury.

Positive BRS is associated with higher risk for development of AKI, e.g. patients with negative BRS and high CRS are at lower risk of developing AKI (category 2) while patients with a positive BRS and low CRS are assigned to category 3 (high risk for the development of AKI) (Table 7) (See Supplementary Notes 1–3 and Supplementary Tables 1–8 for worked examples).

**Table 7:** Proactive AKI clinical tool for management of patients pre- and post-cardiac surgery.

Category	BRS	CRS	Clinical Management
1	Negative	Low	Routine pre or postoperative management
2	Negative	High	Assign to low risk management
3	Positive	Low	Assign to higher risk management
4	Positive	High	Assign to highest risk management

*BRS* biomarker risk score: negative = non AKI, positive = AKI.

*CRS* clinical risk score pre cardiac surgery: low 0–1, high 1.5–3.

*CRS* clinical risk score post cardiac surgery: low 0–4, high 4.5–10.

AKI, acute kidney injury; BRS, biomarker risk score; CRS, clinical risk score.

### Clinical utility; combining BRS with CRS: pre and postoperative management of patients at potential risk for the development of AKI

Combining BRS with CRS could assist with pre and postoperative management of patients at potential risk for the development of AKI. Four categories of risk were identified (Table 7); Categories 1 and 2 = low risk; Categories 3 and 4 = high risk. Combining the biomarkers with the clinical risk factors, preoperative and postoperative, improved the AUROC (See Supplementary Note 4 and Supplementary Table 9 for distribution of non AKI and AKI patients within the risk categories and Supplementary Note 5 and Supplementary Table 10 for further statistical analysis of biomarkers and clinical factors).

## Discussion

The aim of this study was to investigate whether a combination of biomarkers and clinical characteristics/risk score could predict AKI earlier than SCr and oliguria in patients undergoing cardiac surgery. Although a large range of biomarkers were studied, the mediators identified in our model interestingly represented three important pathways for the pathogenesis of renal dysfunction, namely hypoperfusion (H-FABP), ischaemia reperfusion injury (MK) and proinflammatory insult (sTNFR1 or sTNFR2).

Of the n = 30 biomarkers investigated in the patient samples undergoing cardiac surgery, only serum sTNFR1 or sTNFR2 on their own proved to be the best predictive biomarkers pre surgery, whereas serum TNFα was not significant. Soluble TNFR1 and sTNFR2 are the soluble forms of their membrane-bound counterparts (mTNFR1 and mTNFR2) through which TNFα acts^19^. When sTNFR1 and sTNFR2 are released from the membrane, they bind free TNFα thus limiting its biological proinflammatory effects. Soluble TNFR1 and sTNFR2 are thus anti-inflammatory agents^19^. Similarly, postoperative serum sTNFR1 and sTNFR2 had biopredictive utility in combination with MK and H-FABP for AKI whereas TNFα did not.

There are several reasons why this may occur. Firstly, perioperative serum TNFα exhibits different kinetics to serum sTNFR1 and sTNFR2 responses. Serum TNFα has a transient and small increase prior to CPB followed by a second transient and small increase at the end of CPB^20^. These small transient increases may be caused in part, by surgically-induced coagulation disturbances, interaction of blood with the foreign surface of the CPB machine, and retransfusion of unwashed shed mediastinal blood perioperatively^21^. The un-sustained transient nature of the TNFα response reflects efficient mechanisms to clear blood TNFα from the circulation^22^.

Kinetically, unlike TNFα, the serum sTNFR1 and sTNFR2 anti-inflammatory response is larger and more sustained lasting over 24 hours^20^. Moreover, soluble sTNFR2 in blood increases progressively following cardiac surgery over at least a 2-day follow-up period^21^. In this regard, serum sTNFR1 and sTNFR2 responses differ from the blood response of other important anti-inflammatory cytokines such as IL-10 and IL-1Ra which rise and fall to baseline 24 hours perioperative^20^. Furthermore, it may be argued that because the blood IL-10 and IL-1Ra responses at cardiac surgery have been shown to be transient^20^, this may explain why these anti-inflammatory mediators lack biopredictive utility in our model.

The second reason may lie in the underlying pathogenesis of perioperative inflammatory-mediated renal failure. It has been suggested that perioperative increases in filtered TNFα, if unsuccessfully handled by the kidney, could directly injure renal tubules^23^.

Due to the transient nature of TNFα, it is not clinically practicable to measure its exact peak in serum or TNFα recovery from urine^24^. Moreover, serum sTNFR1 and sTNFR2 are >20 kDa and thus not as readily filtered by the tubules as monomeric TNFα. Therefore, increases in blood sTNFR1 and sTNFR2 are not likely to have a direct protective effect against tubular damage mediated by filtered TNFα. This may explain in part why blood increases in sTNFR1 and sTNFR2 were not linked with reduced AKI risk in our model. However, increased blood sTNFR1 and sTNFR2 were linked with AKI risk. This could be because the sustained increases in blood sTNFR1 and sTNFR2 are a proportionate and compensatory response to transient increases in blood TNFα^22^.

As already discussed, serum TNFα is barely detectable in preoperative blood in healthy individuals, whereas baseline preoperative serum sTNFR1 and sTNFR2 concentrations are constitutively expressed^25^. This should be understood in the context of other conditions known to modulate serum sTNFR1 and sTNFR2 levels. For example, both sTNFR1 and sTNFR2 were demonstrated as potential biomarkers for the identification of patients presenting with chronic kidney disease (CKD) by predicting outcome in either those with diabetic nephropathy^26–28^, or early or moderate CKD^29^, or underlying malignancy^30^.

However, in this study we show for the first time that higher baseline sTNFR1 and sTNFR2 in patients who have normal preoperative renal function may predict postoperative AKI risk. Elevated baseline serum sTNFR1 and sTNFR2 preoperatively is driven by a heightened proinflammatory response due to underlying cardiovascular disease processes e.g. atheroma^31^ which would constitute a perioperative AKI risk. Alternatively, a reduced preoperative renal ability to clear preoperative episodic TNFα pulses could lead to a requirement for higher compensatory sustained increased levels in baseline sTNFR1 and sTNFR2.

When the biomarkers (n = 30) were measured in patient serum samples post cardiac surgery at any time the combination of H-FABP, MK and sTNFR1 or sTNFR2 had the highest predictive ability for detecting patients at risk of developing AKI (AUROC 0.817 for H-FABP, MK and sTNFR1 and AUROC 0.836 for H-FABP, MK and sTNFR2 (Table 3)).

While serum sTNFR1 and sTNFR2 in our model may be an indirect reflection of the relative contribution of proinflammatory factors in pathogenesis of AKI, H-FABP in our model may reflect under perfusion of the kidney. Firstly, this increase in serum H-FABP could be secondary to the peri and postoperative myocardial dysfunction which commonly accompanies cardiac surgery^32^. Schaub *et al*.^32^, reported a 6-fold increase in H-FABP measured in blood from patients who experienced AKI at any time point (day 1–day 5 post cardiac operation). Moreover, H-FABP is released into the blood 30 minutes after an ischaemic event from myocytes^33,34^. The resulting suboptimal cardiac output could lead to renal hypoperfusion and AKI. Secondly, H-FABP is also produced by kidney distal tubular cells^35^. However, H-FABP expression in the myocardium is 20 times higher than renal tissue so H-FABP measured in the serum is more likely of myocardial origin^36^. Thirdly, because serum H-FABP is renally cleared, patients with diminished renal function, whether acute or chronic, have compromised H-FABP renal clearance which may further contribute to the elevated H-FABP levels.

Our model also identified MK as a significant factor in the postoperative biomarker combination to detect AKI. Midkine is a pleiotropic, heparin-binding growth factor involved in the pathogenesis of ischemia reperfusion injury. Necrosis and autophagy occur after ischaemic reperfusion injury resulting in vascular endothelial dysfunction and vascular congestion and oedema, reduced blood flow and migration of inflammatory cells to the kidney^37^. Infiltrating inflammatory cells release cytokines, reactive oxygen species (ROS) and other chemokines adding further insult to the already compromised kidney. Midkine promotes this process and is normally expressed at low levels in proximal tubules. However, it is up-regulated in proximal tubules after ischaemic reperfusion^38^. Of note, the absence of MK in MK-deficient mice protects against experimentally induced renal ischaemic reperfusion injury^39^.

The existing method of measuring SCr (eGFR) evaluates the result of AKI. In contrast, our biomarker combination of serum H-FABP, MK and sTNFR1 or sTNFR2, is based largely on the processes initiating and underlying the pathogenesis of AKI. Thus, the information provided by the biomarker combination has the potential to assist with earlier diagnosis and prediction of AKI.

**Figure 4:**
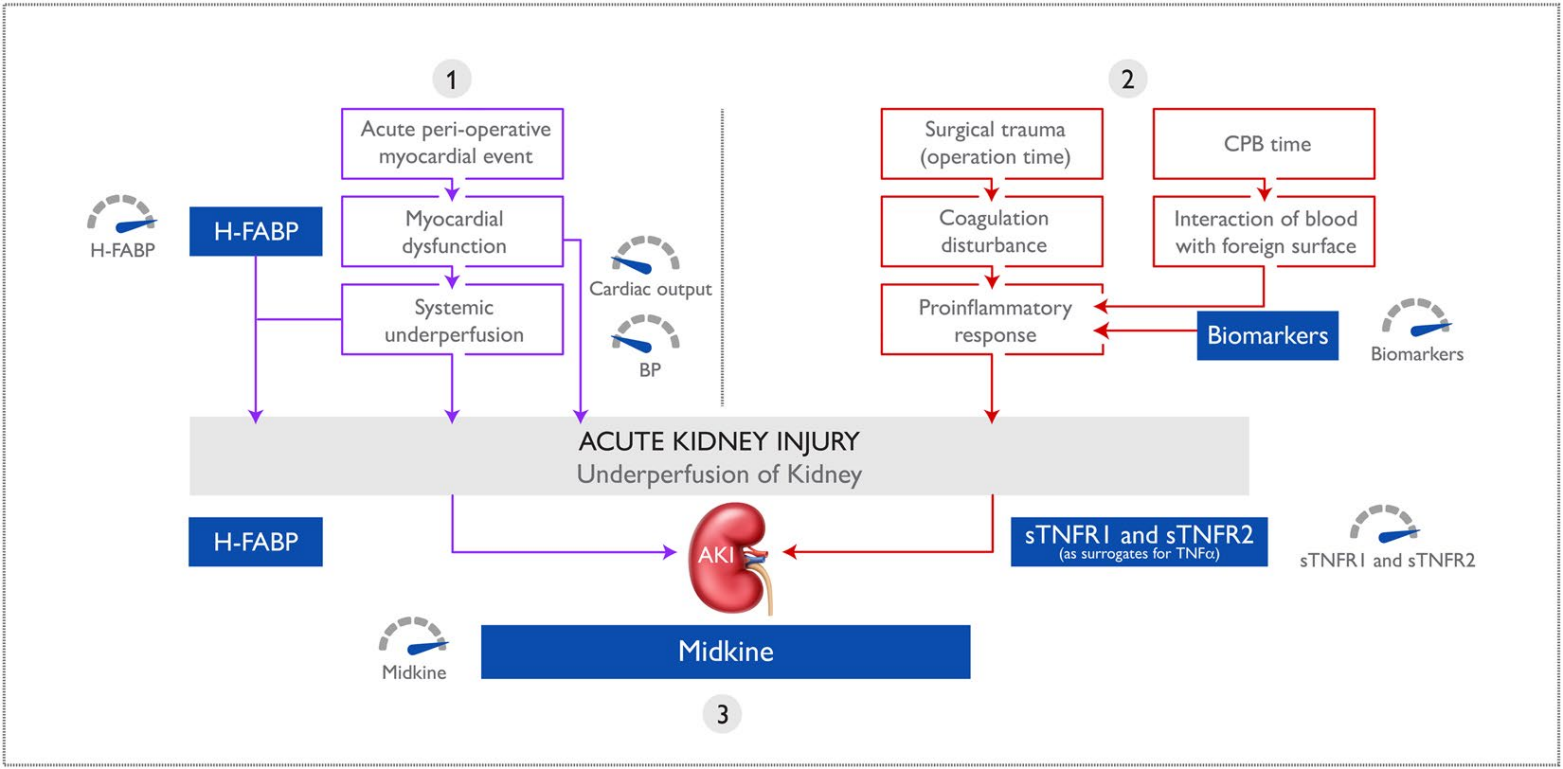
Potential pathways involved in the pathogenesis of AKI. Three important pathways in the pathogenesis of AKI are represented by biomarkers in the model: (1) hypoperfusion (H-FABP), (2) proinflammation (sTNFR1 and sTNFR2 as surrogates for the transient TNFα response) and (3) ischaemia reperfusion injury (MK). Together with clinically measured variables, such as (among others) cardiac output and blood pressure (hypoperfusion and ischaemia reperfusion), cross clamp time and bypass time (proinflammation) biomarkers enable AKI patient risk categorisation. AKI, acute kidney injury; H-FABP, heart-type fatty acid-binding protein; sTNFR1, soluble tumour necrosis factor receptor 1; sTNFR2, soluble tumour necrosis factor receptor 2; TNFα, tumour necrosis factor alpha; MK, midkine.

In summary, three main factors in perioperative AKI at cardiac surgery, namely proinflammatory-mediated tubular injury, renal under perfusion and ischemia reperfusion injury are utilised in our model. Soluble TNFR1 and sTNFR2 indicate perioperative proinflammatory load, H-FABP indicates the risk of renal under perfusion secondary to myocardial dysfunction, and MK suggests renal ischemia reperfusion injury. A potential mechanism of action for the biomarker combination is described in Fig. 4.

The onset of AKI is multifactorial so in addition to biomarkers, clinical characteristics including age, BMI and diabetes were identified as risk factors for patients at potential risk for the development of AKI preoperatively (Table 4). These three clinical factors were also identified together with surgery-related factors, which included CPB time, cross-clamp time, operation time, whether the patient needed intra-aortic balloon pump, transfusion of blood or platelets and resternotomy, for identifying AKI in patients postoperatively (Table 5). To translate both the biomarker data and clinical characteristics into a proactive AKI clinical tool, the information was converted into a BRS and CRS, respectively (Tables 4, 5, 6 and 7). The results from the BRS and CRS combination will allow the clinician to identify patients at risk of AKI, administer appropriate treatments and monitor treatment efficacy. Thus, if a patient was identified as risk category 2, the current author (WMcB) would monitor the patient’s expected increases in creatinine and urea concentrations over several days retaining an expectation that dialysis requirement would be unlikely. A diuretic, if necessary, would be considered. If the patient was identified as category 3, the dialysis machine would be made available but not primed for potential use post surgery. The present author would be more hesitant to give a diuretic to this patient. However, if the patient was identified as category 4, the author would request that the dialysis machine was ready for use once the operation was completed.

The patients who developed AKI in the current study stayed an extra 2 days in hospital and 2 days longer in ICU (p = 0.000 and p = 0.000, respectively). Similarly, AKI patients had significant increased length of stay in the HDU (p = 0.001). Additional hospital stay is associated with increased costs. However, the low risk patients could potentially have been moved out of the HDU to a ward which would improve patient flow and free up beds and staff to accept new patients, with associated savings (2018/2019 costs per day in HDU is £1400 (excluding medication))^40^. Earlier diagnosis of AKI benefits the patient, clinician and improves use of hospital resources.

### Limitations of the study

Patients undergoing cardiac surgery were included in the study and, therefore, AKI resulting from other serious diseases such as sepsis or drug-induced AKI, were not represented. This was an observational study, where biomarker analysis was completed post event. This limits conclusions since patient interventions were not influenced by our results. Strengths of the study include; the patients were considered not renally impaired preoperatively which enabled measurement of baseline biomarkers. This assisted with determination of biomarker levels post surgery and an understanding of the role of biomarkers in AKI development.

## Conclusion

Measurement of sTNFR1 or sTNFR2 preoperatively predicted risk of a patient developing AKI following cardiac surgery. Measurement of a combination of biomarkers, namely H-FABP, MK, sTNFR1 or sTNFR2, at any time postoperatively identified patients with increased risk of developing AKI. Furthermore, deployment of a BRS in combination with CRS in routine practice could assist the clinician with appropriate patient management. This would allow identification of patients at higher risk of developing AKI pre and immediately postoperatively. Adoption of this novel proactive AKI clinical tool will (1) facilitate early identification of patients at risk of AKI, (2) allow timelier clinical decision-making, (3) alter current patient pathways, resulting in more efficient hospital resources utilisation and reduced hospital/ICU/HDU stay.

## Supplementary information

Supplementary Information

## Data Availability

The datasets used and/or analysed during the current study are available from the corresponding author on reasonable request.
