# Supplementary material for: Stratifying risk of acute kidney injury in pre and post cardiac surgery patients using a novel biomarker-based algorithm and clinical risk score
Source: Sci Rep. 2019 Nov 18;9:16963. doi: 10.1038/s41598-019-53349-1 (PMC6861253; doi:10.1038/s41598-019-53349-1)
Supplement: Supplementary file 1 — Supplementary Information [file 41598_2019_53349_MOESM1_ESM.pdf]

Supplementary information

Stratifying risk of acute kidney injury in pre and post cardiac surgery patients using a novel biomarker-based algorithm and clinical risk score

Authors: William T. McBride, Mary Jo Kurth, Gavin McLean, Anna Domanska, John V. Lamont, Daniel Maguire, Joanne Watt, Peter Fitzgerald, Ian Young, Jijin Joseph and Mark W. Ruddock.

Supplementary Note 1. Worked Examples

Two patients, one non AKI and one with AKI, were randomly chosen from the database. The data from each patient pre and post cardiac surgery was applied to the BRS and CRS to demonstrate the clinical utility in routine practice.

Even though the CRS was high in the non AKI patient pre and postoperatively, the BRS was negative, therefore, the patient would be placed in category 2 "low risk management".

In the AKI patient BRS was positive pre and postoperatively. Preoperative CRS was high due to age, obesity and diabetes (3/3) and the postoperative CRS put the patient into a low risk category (4/10). This patient was assigned to category 4 pre surgery and category 3 post surgery. A category 4 assignment pre surgery allows surgical strategy to be modified to reduce cross clamp and bypass times. For example, in a preoperative category 4, a surgeon may decide to deploy a sutureless aortic valve which substantially reduces cross clamp time.

Supplementary Note 2. Worked example BRS and CRS for patient C313 (non AKI)

Preoperative:

BRS
sTNFR1 - Negative (non AKI)

Supplementary Table S1. CRS

Clinical Factors	Parameter	Result
Age = 74 years	<65<br>**>65**	0<br>**1**
BMI = 31.02	<25<br>>25 <30<br>**>30**	0<br>0.5<br>**1**
Diabetes = No	**No**<br>Yes	**0**<br>1
	**Total**	**2**/3

Postoperative:

BRS
H-FABP + MK + sTNFR2 - Negative (non AKI)

Supplementary Table S2. CRS

Clinical Factors	Parameter	Result
Age = 74 years	<65<br>**>65**	0<br>**1**
BMI = 31.02	<25<br>>25 <30<br>**>30**	0<br>0.5<br>**1**
Diabetes = No	**No**<br>Yes	**0**<br>1
CPB time = 171 min	<130<br>**>130**	0<br>**1**
Cross clamp time = 123 min	<90<br>**>90**	0<br>**1**
Operation time = 300 min	<296<br>**>296**	0<br>**1**
Intra-aortic balloon pump = 0	**No**<br>Yes	**0**<br>1
Packed red blood cells = 1	No<br>**Yes**	0<br>**1**
Platelet bags = 0	**No**<br>Yes	**0**<br>1
Resternotomy = 0	**No**<br>Yes	**0**<br>1
	Total	**6**/10

Supplementary Table S3. Preoperative BRS and CRS:

Category	BRS	CRS	Clinical Management
1	Negative	Low	Routine pre or postoperative management
**2**	**Negative**	**High**	**Assign to low risk management**
3	Positive	Low	Assign to higher risk management
4	Positive	High	Assign to highest risk management

Supplementary Table S4. Postoperative BRS and CRS:

Category	BRS	CRS	Clinical Management
1	Negative	Low	Routine pre or postoperative management
**2**	**Negative**	**High**	**Assign to low risk management**
3	Positive	Low	Assign to higher risk management
4	Positive	High	Assign to highest risk management

Supplementary Note 3. Worked example BRS and CRS for patient C079 (AKI)

Preoperative:

BRS
sTNFR1 - Positive (AKI)

Supplementary Table S5. CRS

Clinical Factors	Parameter	Result
Age = 78 years	<65<br>**>65**	0<br>**1**
BMI = 31.37	<25<br>>25 <30<br>**>30**	0<br>0.5<br>**1**
Diabetes = Yes	No<br>**Yes**	0<br>**1**
	**Total**	**3**/3

Postoperative:

BRS
H-FABP + MK + sTNFR2 - Positive (AKI)

Supplementary Table S6. CRS

Clinical Factors	Parameter	Result
Age = 78 years	<65<br>**>65**	0<br>**1**
BMI = 31.37	<25<br>>25 <30<br>**>30**	0<br>0.5<br>**1**
Diabetes = Yes	No<br>**Yes**	0<br>**1**
CPB time = 118 min	**<130**<br>>130	**0**<br>1
Cross clamp time = 85 min	**<90**<br>>90	**0**<br>1
Operation time = 240 min	**<296**<br>>296	**0**<br>1
Intra-aortic balloon pump = 0	**No**<br>Yes	**0**<br>1
Packed red blood cells = 1	No<br>**Yes**	0<br>**1**
Platelet bags = 0	**No**<br>Yes	**0**<br>1
Resternotomy = 0	**No**<br>Yes	**0**<br>1
	Total	**4**/10

Supplementary Table S7. Preoperative BRS and CRS:

Category	BRS	CRS	Clinical Management
1	Negative	Low	Routine pre or post operative management
2	Negative	High	Assign to low risk management
3	Positive	Low	Assign to higher risk management
**4**	**Positive**	**High**	**Assign to highest risk management**

Supplementary Table S8. Postoperative BRS and CRS:

Category	BRS	CRS	Clinical Management
1	Negative	Low	Routine pre or postoperative management
2	Negative	High	Assign to low risk management
**3**	**Positive**	**Low**	**Assign to higher risk management**
4	Positive	High	Assign to highest risk management

Supplementary Note 4. Distribution of non AKI and AKI patients within risk categories 1-4

The risk of developing AKI was low for patients who were categorised either 1 or 2. Whereas patients categorised 3 or 4 were at higher risk for developing AKI. If the BRS was negative, patients were categorised either 1 or 2. A combination of BRS and CRS categorised >90% of non AKI patients in category 1 and almost 80% in category 2. Thus, a combination of BRS and CRS identified cardiac patients that were at lower risk of developing AKI and assigned them to the low risk clinical management category. Patients with positive BRS were assigned to either category 3 or 4, high risk.

Supplementary Table S9. Postoperative BRS (H-FABP + MK + sTNFR2) and CRS

	**Category 1**	**Category 2**	**Category 3**	**Category 4**	**Total**
**Non AKI**	103 (98.2%)	46 (79.3%)	21 (60.0%)	17 (50.0%)	187 (78.6%)
**AKI**	8 (7.2%)	12 (20.7%)	14 (40.0%)	17 (50.0%)	54 (21.4%)
**Total**	111 (100%)	58 (100%)	35 (100%)	34 (100%)	238 (100%)

Figures in table refer to number and percentage of patients within each risk category identified as non AKI or AKI using a combination of BRS and CRS

Supplementary Note 5. Combining biomarkers and clinical risk factors for AKI

Preoperative clinical risk factors (age, BMI and diabetes) when combined with biomarkers for AKI significantly increase the AUROC

Preoperatively

Soluble TNRF1 and TNFR2 had the highest predictive ability to identify patients as risk of AKI (AUC 0.748 and AUC 0.713, respectively). Age, BMI and diabetes were identified as preoperative clinical risk factors (age AUC 0.599, BMI AUC 0.625, and diabetes AUC 0.564). Combining age[a], BMI[a] and diabetes[a] gave a model with an AUC 0.680. However, when the clinical risk factors were combined with the biomarker(s), only BMI was significant i.e. sTNFR1 + BMI AUC 0.761 and sTNFR2 + BMI AUC 0.736.

Postoperative clinical risk factors (age, BMI, diabetes, CPB, cross clamp time, operation time, intra-aortic balloon pump, packed RBCs, platelets and resternotomy) when combined with biomarkers for AKI significantly increase the AUROC

Postoperatively

In combination, midkine, sTNFR1, and H-FABP or midkine, sTNFR2 and H-FABP had the highest predictive ability to identify patients at risk of AKI (AUC 0.817 and AUC 0.836, respectively). Age[b], BMI[b], diabetes[b], CPB[b], cross-clamp time[b], operation time[b], intra-aortic balloon pump[b], packed RBCs[b], platelets[b] and resternotomy[b] were identified as preoperative clinical risk factors (age AUC 0.599, BMI AUC 0.625, diabetes AUC 0.564, CPB AUC 0.551, cross clamp time AUC 0.586, operation time AUC 0.582, intra-aortic balloon pump AUC 0.535, packed RBCs AUC 0.569, platelets AUC 0.513, and resternotomy AUC 0.557). When clinical risk factors were combined, only age, BMI, diabetes and operation time were significant AUC 0.703. Combining all clinical risk factors with midkine + sTNFR1 or sTNFR2, and H-FABP identified the following: (1) midkine + sTNFR1 + H-FABP + BMI AUC 0.833; (2) midkine + sTNFR2 + H-FABP + BMI + CPB + cross-clamp time AUC 0.881. Surprisingly, only BMI was

significant in the post-operative model that included sTNFR1. Whereas, BMI, CBP and cross-clamp time were significant in the post-operative model that included sTNFR2.

Supplementary Table S10.

Pre or Post Surgery	Biomarker(s) and/or clinical factor(s)	AUROC	PPV (%)	NPV (%)
Pre	sTNFR1	0.748	35.4	90.4
	sTNFR2	0.713	33.9	88.9
	Age, BMI, Diabetes	0.680	27.7	87.5
	sTNFR1 + BMI[a]	0.761	38.8	90.1
	sTNFR2 + BMI[a]	0.738	34.3	88.3
Post	sTNFR1 + MK + H-FAPB	0.817	44.2	92.6
	sTNFR2 + MK + H-FAPB	0.836	41.9	93.9
	Age + BMI + diabetes + operation time	0.703	32.4	90.2
	sTNFR1 + MK + H-FAPB + BMI[b]	0.833	43.6	92.7
	sTNFR2 + MK + H-FAPB + BMI + CPB + cross-clamp time[b]	0.881	54.7	93.9

[a]Preoperative clinical risk factors age, BMI, and diabetes were imputed as categorical variables. However, only BMI was significant in the biomarker-based algorithms.

[b]Postoperative clinical risk factors age, BMI, diabetes, CPB, cross-clamp time, operation time, intra-aortic balloon pump, packed RBCs, platelets and resternotomy were also imputed as categorical variables. However, only BMI, CPB and cross-clamp time were significant in the biomarker-based algorithms.

AUROC, area under receiver operating characteristic; PPV, positive predictive value; NPV, negative predictive value; sTNFR1, soluble tumour necrosis factor 1; sTNFR2, soluble tumour necrosis factor 2; MK, midkine; H-FABP, heart-type fatty acid-binding protein; BMI, body mass index; CPB, cardio pulmonary bypass.
